# Invasive Thymoma with Right Upper Lobe Endobronchial Lesion and Autoimmune Enteropathy

**DOI:** 10.1155/2020/6396915

**Published:** 2020-06-20

**Authors:** Saumil Datar, Henriette De La Garza, Aditya Srinivasan, Gloria Iliescu, Neda Kalhor, Horiana Grosu

**Affiliations:** ^1^McGovern Medical School, The University of Texas MD Anderson Cancer Center, Houston, Texas, USA; ^2^Instituto Tecnologico y de Estudios Superiores de Monterrey, Monterrey, Mexico; ^3^Department of Pulmonary Medicine, The University of Texas MD Anderson Cancer Center, Houston, Texas, USA; ^4^Department of Internal Medicine, The University of Texas MD Anderson Cancer Center, Houston, Texas, USA; ^5^Department of Cytopathology, The University of Texas MD Anderson Cancer Center, Houston, Texas, USA

## Abstract

Thymomas are slow-growing neoplasia arising from the epithelial cells of the thymus that usually present with respiratory symptoms, superior vena cava syndrome, or parathymic syndromes. Approximately 30% of thymomas develop myasthenia gravis. An additional 5% of patients with thymomas have other systemic syndromes, including rheumatoid arthritis, thyroiditis, red cell aplasia, systemic lupus erythematosus, and Cushing syndrome. Rarely, patients can present with diarrhea due to thymoma-associated autoimmune gastrointestinal pathologies that include Good syndrome (acquired hypogammaglobulinemia), thymoma- associated multiorgan autoimmunity, and autoimmune enteropathy. We present an uncommon and interesting case of an invasive metastatic thymoma with right upper lobe endobronchial lesion and autoimmune enteropathy in a 27-year-old female. The novelty of this case lay in the findings of extensive metastatic thymoma with right upper lobe endobronchial disease and autoimmune diarrhea.

## 1. Introduction

Thymomas originate within the epithelial cells of the thymus and are the most common neoplasm of the anterior mediastinum, representing 20% of all mediastinal tumors and 50% of anterior mediastinal masses. Its peak incidence occurs in the fourth and fifth decades of life. The etiology of thymomas has not been established; however, thymomas are associated with several paraneoplastic syndromes. Approximately 30% of thymomas develop myasthenia gravis. An additional 5% of patients with thymomas have other systemic syndromes, including rheumatoid arthritis, thyroiditis, red cell aplasia, systemic lupus erythematosus, and Cushing syndrome.

Malignant thymomas can invade the vasculature, lymphatics, and adjacent structures within the mediastinum. The 15-year survival rate is 12.5% for a person with an invasive thymoma and 47% for a person with a noninvasive thymoma. Death usually occurs from cardiac tamponade or other cardiorespiratory complications [1].

## 2. Case

**Figure 1:**
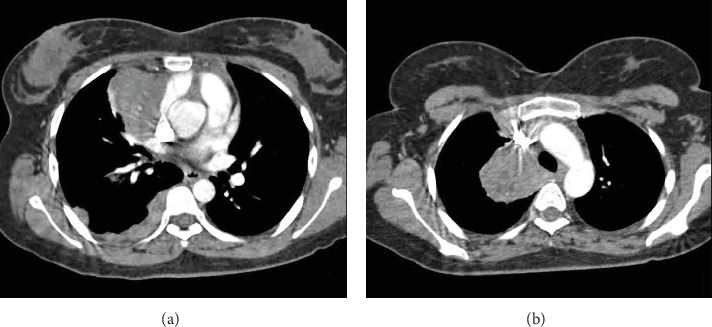
(a) Computed tomography image of the chest depicting a large right upper paratracheal mass. (b) Computed tomography image of the chest depicting pleural and pericardial.

**Figure 2:**
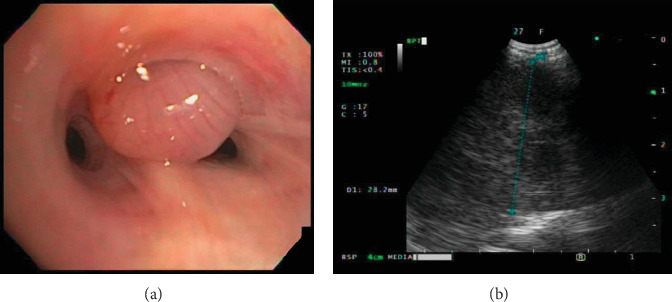
(a) Endobronchial smooth polypoid lesion arising from the right upper lobe apical segment. (b) Endobronchial ultrasound image of the right paratracheal heterogeneous mass.

**Figure 3:**
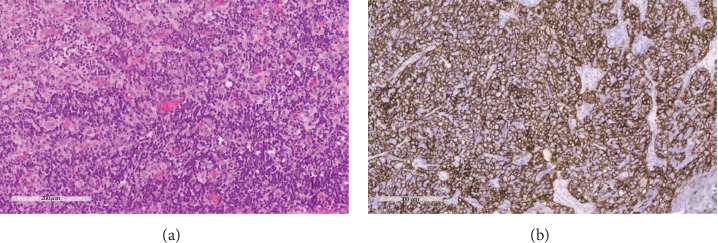
(a) High-magnification image showing a biphasic neoplasm composed of lymphocytes and epithelial cells. (b) Immunostaining for high molecular weight cytokeratin (CK5/6) highlighting the epithelial component. The lymphocytes are negative.

A 27-year-old woman with no history of malignancy presented to our hospital with chronic watery diarrhea of 6 months duration. She denied any respiratory symptoms. All infectious disease workup was negative. Upper gastrointestinal tract endoscopy and colonoscopy were negative for pertinent findings. A computed tomography study of the abdomen was normal but showed several right lung base masses. A subsequent computed tomography study of the chest revealed extensive right lung metastatic disease and a large lobulated right upper paratracheal mass (Figures 1(a) and 1(b)). The initial differential diagnoses included lymphoma and thymoma. Transthoracic biopsy was negative for malignancy, but flow cytometry was consistent with a T-cell population of thymic origin. Bronchoscopy with endobronchial ultrasound/ultrasound-guided transbronchial needle aspiration was performed for further evaluation. Endobronchial ultrasound revealed a large heterogeneous paratracheal mass and a polypoid smooth endobronchial lesion in the apical segment of the right upper lobe (B1; Figures 2(a) and 2(b)). Pathology from the endobronchial lesion was consistent with metastatic thymoma (Figures 3(a) and 3(b)). She was started on treatment with cisplatin/prednisone/cyclophosphamide/doxorubicin; however, due to unforeseen circumstances, she was lost to follow-up.

## 3. Discussion

Thymomas are slow-growing neoplasia arising from the epithelial cells of the thymus that usually present with respiratory symptoms, superior vena cava syndrome, or parathymic syndromes. An autoimmune process follows in up to 40% of patients with thymoma, and half of these patients develop subsequent myasthenia gravis [2]. Patients can present with diarrhea due to thymoma-associated autoimmune gastrointestinal pathologies that include Good syndrome (acquired hypogammaglobulinemia), thymoma-associated multiorgan autoimmunity, and autoimmune enteropathy [3]. The specific cause of our patient's diarrhea was thought to be autoimmune enteropathy. Endobronchial disease has rarely been reported in the literature, and most cases involved invasion of the left upper bronchus, especially the left B3 bronchus, and expansion of the bronchial lumen [4].

The novelty of this case lay in the findings of extensive metastatic thymoma with right upper lobe endobronchial disease and autoimmune diarrhea in a very young patient. Thymomas typically account for about half (47%) of anterior mediastinal tumors [5]. Malignant thymomas, however, account for less than 0.5% of all malignancies and thymic carcinoma accounts for 5% of thymic malignancies [5]. The WHO classification distinguishes thymomas from thymic carcinomas based on the morphology of epithelial tumor cells, proportion of lymphocytic involvement, and resemblance to normal thymic tissue. In addition, thymomas present with variety of autoimmune disorders as opposed to patients with thymic carcinoma who rarely will have autoantibody-induced phenomena [6]. Much of our knowledge on the treatment and management of these conditions stems from case reports, which frequently show extensive lung or mediastinal invasion at initial presentation in newly diagnosed patients. There is no consensus on the best approach in managing metastatic thymic disease; surgery continues to be the mainstay of treatment and complete resection of the tumor remains the most important prognostic factor.

Multimodality therapy seems an appropriate approach to stage III and IV thymomas [7]. Our patient was started on treatment with cisplatin/prednisone/cyclophosphamide/doxorubicin. In a patient who presents with chronic diarrhea and is found to have mediastinal masses, thymoma should be considered in the differential diagnosis and an immediate autoimmune workup should be done to determine optimal medical management.

## References

[1] Bushan K., Sharma S., Verma H. (2013). A review of thymic tumors. *Indian Journal of Surgical Oncology*.

[2] Okumura M., Fujii Y., Shiono H. (2008). Immunological function of thymoma and pathogenesis of paraneoplastic myasthenia gravis. *General Thoracic and Cardiovascular Surgery*.

[3] Joven M. H., Palalay M. P., Sonido C. Y. (2013). Case report and literature review on Good’s syndrome, a form of acquired immunodeficiency associated with thymomas. *Hawai'i Journal of Medicine & Public Health*.

[4] Abiko M., Sato T., Shiono S. (1999). A case of invasive thymoma displaying endobronchial extension. *Kikansigaku*.

[5] Detterbeck F. C., Zeeshan A. (2013). Thymoma: current diagnosis and treatment. *Chinese Medical Journal*.

[6] Margaritora S., Cesario A., Cusumano G. (2010). Thirty-five-year follow-up analysis of clinical and pathologic outcomes of thymoma surgery. *The Annals of Thoracic Surgery*.

[7] D’Andrea M. A., Reddy G. K. (2015). Management of metastatic malignant thymoma with advanced radiation and chemotherapy techniques: report of a rare case. *World Journal of Surgical Oncology*.

